# The complete compositional epistasis detection in genome-wide association studies

**DOI:** 10.1186/1471-2156-14-7

**Published:** 2013-02-19

**Authors:** Xiang Wan, Can Yang, Qiang Yang, Hongyu Zhao, Weichuan Yu

**Affiliations:** 1 Department of Computer Science and Institute of Theoretical and Computational Study, Hong Kong Baptist University; 2 Department of Biostatistics, Yale School of Public Health, New Haven, Connecticut 06520, USA; 3 Department of Computer Science and Engineering, The Hong Kong University of Science and Technology, Clear Water Bay, Kowloon, Hong Kong, China; 4 Department of Electronic and Computer Engineering, The Hong Kong University of Science and Technology, Clear Water Bay, Kowloon, Hong Kong, China

**Keywords:** Compositional epistasis, SNP, Genome-wide association study, GPU

## Abstract

**Background:**

The detection of epistasis among genetic markers is of great interest in genome-wide association studies (GWAS). In recent years, much research has been devoted to find disease-associated epistasis in GWAS. However, due to the high computational cost involved, most methods focus on specific epistasis models, making the potential loss of power when the underlying epistasis models are not examined in these analyses.

**Results:**

In this work, we propose a computational efficient approach based on complete enumeration of two-locus epistasis models. This approach uses a two-stage (screening and testing) search strategy and guarantees the enumeration of all epistasis patterns. The implementation is done on graphic processing units (GPU), which can finish the analysis on a GWAS data (with around 5,000 subjects and around 350,000 markers) within two hours. Source code is available at http://bioinformatics.ust.hk/BOOST.htmlâˆ–#GBOOST.

**Conclusions:**

This work demonstrates that the complete compositional epistasis detection is computationally feasible in GWAS.

## Background

The concept of epistasis was first introduced in 1909 by Bateson and Mendel [1] to describe the masking effect of one locus over another locus. Today, it is broadly referred to as joint effects across different genes on phenotypes. The identifications of epistasis between two loci can offer insights on the complex biological pathways underlying human diseases [2]. With genome-wide genotyping microarrays, it is possible to evaluate epistasis at the genomic level through the analysis of genome-wide association studies (GWAS) where hundreds or thousands of subjects are genotyped at up to millions of single nuclear polymorphisms (SNPs). Because epistasis can involve markers with or without significant marginal effects [3-5], a comprehensive investigation of epistasis is a necessary step following the traditional single marker analysis in finding susceptibility markers of complex diseases. However, hundreds of billions of SNP pairs need to be considered if an exhaustive search is conducted and the significant computational cost has restrained researchers from conducting a full investigation of epistasis in GWAS.

Researchers generally distinguish three types of epistasis: functional epistasis, statistical epistasis, and compositional epistasis [6,7]. Functional epistasis indicates molecular interactions in the biological context. Statistical epistasis [8] defines the joint behavior of two loci as the statistical deviation from their additive effects. Compositional epistasis maintains the original concepts given by Bateson and Mendel [1], which can be interpreted as two-locus epistasis models (see details in the Methods Section).

Estimating statistical epistasis between two loci requires the estimation of their additive main effects, which involves iterations (see details in the Methods Section). Because hundreds of billions of SNP pairs need to be measured for epistasis in a standard GWAS, any extra time spent on analyzing each pair will significantly increase the computational cost. To tackle this computational problem, many earlier methods [9-12] used a heuristic procedure that first removes all significant loci based on single-locus tests and then computes the statistical epistasis of two loci with the sum of individual effects and interaction effects. Recently, [3] developed a non-iterative method to approximate the likelihood ratio statistic, which make the detection of pure statistical epistasis (only the interaction effect) computationally feasible in GWAS. However, all the methods mentioned above may suffer from the issue where the underlying degree of freedom is lower than the one assumed in their statistical tests. This issue is mainly caused by the low minor allele frequency (MAF) of loci, which leads to the sparse contingency table in the test. To solve this issue, one solution is to test the compositional epistasis.

It has been argued that compositional epistasis is closer to the biological understanding of gene-gene interactions than statistical epistasis [6]. However, for each pair of loci, there are 512 epistatic patterns defined by compositional epistasis. There is a heavy computational burden in analyzing GWAS data if all these patterns are considered. To our knowledge, there is no method to find compositional epistasis in GWAS data.

In this article, we propose a fast approach to enable exhaustive search of compositional epistasis in GWAS. The proposed approach uses a two-stage (screening and testing) search strategy. In the screening stage, only a limited number of epistatic patterns are evaluated for each pair of SNPs and those passing a specified threshold are selected. All non-significant pairs are filtered out and those pairs, which are significant in the test of compositional epistasis, will be kept in the remaining set. In the testing stage, we evaluate all epistatic patterns for each remaining pair. The implementation is done on graphic processing units (GPU), where the analysis of one GWAS data set (with around 5,000 subjects and around 350,000 markers) can be finished within a few hours.

## Methods

SNPs are mostly bi-allelic genetic markers. In general, we use capital letters (e.g., A, B, ⋯) to denote the major alleles and lowercase letters (e.g., a, b, ⋯) to denote the minor alleles. For each SNP, there are three genotypes: the homozygous reference genotype (AA), the heterozygous genotype (Aa), and the homozygous variant genotype (aa). The popular way of coding the genotype is to use {1, 2, 3} to represent {*AA*,*Aa*,*aa*}, respectively.

### Epistasis tests

The statistical epistasis and the compositional epistasis are two major types of epistasis that have been considered in the literature. The statistical epistasis is defined as the statistical deviation from the additive effects of two loci on the phenotype [8]. One popular way to test the statistical epistasis is to use the likelihood ratio test. Given two SNPs *X*_*p*_ and *X*_*q*_, there are three steps in such a procedure: 

• Fit the logistic regression model for only individual effect terms and obtain the MLE ${\hat{L}}_{M}$

(1) $$\log\frac{P(Y=1|X_{p},X_{q})}{P(Y=2|X_{p},X_{q})}=\beta_{0}+\beta_{i}^{X_{p}}+\beta_{j}^{X_{q}}.$$

• Fit the logistic regression model for both individual effect terms and interaction terms and obtain the MLE ${\hat{L}}_{F}$

(2) $$\log\frac{P(Y=1|X_{p},X_{q})}{P(Y=2|X_{p},X_{q})}=\beta_{0}+\beta_{i}^{X_{p}}+\beta_{j}^{X_{q}}+\beta_{\text{ij}}^{X_{p}X_{q}}.$$

• Conduct the *χ*^2^ test on $2\cdot ({\hat{L}}_{F}-{\hat{L}}_{M})$ with *df* = 4.

We call this test as interaction test. However, estimating the MLE ${\hat{L}}_{M}$ involves iterations (the estimation of the MLE ${\hat{L}}_{F}$ has the closed-form solution), which is computationally very expensive to evaluate hundreds of billions of pairs in GWAS. Therefore, many methods use a different procedure to estimate the epistasis. 

• Remove all significant SNPs based on the single-locus test with a given threshold.

• For every pair (*X*_*p*_,*X*_*q*_) in the remaining SNPs, 

– Compute the log-likelihoods *L*_*∅*_ of the null logistic regression model, defined as

(3) $$\log\frac{p}{1-p}=\beta_{0}.$$

– Compute the log-likelihoods *L*_*F*_ of the full logistic regression model in Eq.(2).

– Conduct *χ*^2^ tests on 2·(*L*_*F*_−*L*_*∅*_) with 8 degrees of freedom.

We call the test with 8 degrees of freedom as full association test. In the full association test, a threshold is required to filter out the significant SNPs. Otherwise, it will produce many false epistasis involving one marginally significant SNP with an irrelevant one.

The full association test is totally different from the interaction test. It measures the sum of individual effects and interaction effects and thus its degrees of freedom is 8 while the interaction test only only measures the interaction effect with 4 degrees of freedom. Both tests have their pros and cons. In the full association test, it is very difficult to decide the threshold to filter out the significant SNPs. For a stringent threshold, many SNPs below the threshold may produce strong associations in the full model with a little interaction effect. For a loose threshold, some SNPs involved in true epistasis may be filtered out. In the interaction test, those epistasis involving SNPs having medium individual effects and meanwhile having medium interaction effect will be ignored. Most importantly, they all suffer from the issue where the underlying degree of freedom is lower than the one assumed in their statistical tests, which is caused by the low MAF. The relatively robust solution to tackle this issue is to use the test of compositional epistasis.

### The definition of two-locus compositional epistasis

A two-locus compositional epistasis can be defined by a 3-by-3 penetrance table (see Table 1). The columns represent the three genotypes of the first SNP and the rows represents the three genotypes of the second SNP. The entry *p*_*ij*_ in this table is the probability of developing a disease with the corresponding joint genotype at the two SNPs. One common approach of defining disease models is to restrict the value of *p*_*ij*_ to two levels, e.g., 0 or 1, which corresponds low risk or high risk. With this restriction, the total number of possible epistasis patterns is 2^9^=512. Each model can be associated with a unique label which is defined as the decimal number of (*p*_11_*p*_12_*p*_13_*p*_21_*p*_22_*p*_23_*p*_31_*p*_32_*p*_33_)_2_. For example, Table 2 gives the definition of popular dominant epistasis model. The label of the dominant epistasis model is (000011011)_2_=27. Because of the symmetry in the model definition, the number of non-redundant epistasis models is less than 512. In [13], it was shown that there are 51 unique epistatic patterns, including the recessive model (M1) and some complicated patterns which may be difficult to interpret biologically (e.g., M170).

**Table 1:** Two locus penetrance table

	***S N P***_**2**_**=1**	***S N P***_**2**_**=2**	***S N P***_**2**_**=3**
*SNP*_1_=1	*p*_11_	*p*_12_	*p*_13_
*SNP*_1_=2	*p*_21_	*p*_22_	*p*_23_
*SNP*_1_=3	*p*_31_	*p*_32_	*p*_33_

The element ***p***_***ij***_ is the probability of developing a disease with the corresponding joint genotype at the two SNPs.

**Table 2:** The dominant epistasis model

	***S N P***_**2**_**=1**	***S N P***_**2**_**=2**	***S N P***_**2**_**=3**
*SNP*_1_=1	0	0	0
*SNP*_1_=2	0	1	1
*SNP*_1_=3	0	1	1

Its unique label is 27=(000011011)_2_.

The trivial *p*_*ij*_ in Table 1 will decrease the power of both the full association test and the interaction test. The compositional epistasis can solve this issue by reducing the 3×3 penetrance table into the 2×2 risk table according to the model definition.

### The test of two-locus compositional epistasis

To identify the compositional epistasis for *SNP*_*i*_ and *SNP*_*j*_, a contingency table of these two SNPs and the class label *Y* should be collected first (See Table 3). The size of the contingency table is 3×3×2. In Table 3, *n*_*ijk*_ denotes the observed count in the cell (*i*,*j*,*k*). The total number of samples is $n=\underset{i,j,k}{\sum }n_{i,j,k}$.

**Table 3:** **The genotype counts in controls (*Y *= *0*) and cases (*Y *= *1*)**

***Y *= 0**	***S N P***_***j ***_**= 1**	***S N P***_***j ***_**= 2**	***S N P***_***j ***_**= 3**	***Y *= 1**	***S N P***_***j ***_**= 1**	***S N P***_***j ***_**= 2**	***S N P***_***j ***_**= 3**
*SNP*_*i *_= 1	*n*_110_	*n*_120_	*n*_130_	*SNP*_*i *_= 1	*n*_111_	*n*_121_	*n*_131_
*SNP*_*i *_= 2	*n*_210_	*n*_220_	*n*_230_	*SNP*_*i *_= 2	*n*_211_	*n*_221_	*n*_231_
*SNP*_*i *_= 3	*n*_310_	*n*_320_	*n*_330_	*SNP*_*i *_= 3	*n*_311_	*n*_321_	*n*_331_

Next, for a particular compositional epistasis model defined by a penetrance table, Table 3 can be converted into a 2×2 risk table (See Table 4). For example, for the dominant epistasis model defined in Table 2, the risk table is defined with *a*=*n*_110_+*n*_120_+*n*_130_+*n*_210_+*n*_310_, *b*=*n*_220_+*n*_230_+*n*_320_+*n*_330_, *c*=*n*_111_+*n*_121_+*n*_131_+*n*_211_+*n*_311_, and *d*=*n*_221_+*n*_231_+*n*_321_+*n*_331_. The risk table allows us to compare the proportion of samples in cases and controls with the assumption that the given epistasis model is true. If the proportions of samples in different rows vary significantly between columns, we draw a conclusion that the risk factors (genotypes) and the disease traits (class labels) are not independent for the given epistasis model. The significance of the difference between the two proportions can be assessed with Pearson’s chi-squared test. The test statistic is defined in Eq.(4) with the degree of freedom *df* = 1.

(4) $$X_{\text{df}=1}^{2}=\frac{{\left(\text{ad}-\text{bc}\right)}^{2}(a+b+c+d)}{\left(a+b)(c+d)(a+c)(b+d\right)}$$

**Table 4:** Risk table for testing the fit of an epistasis model

	**Low risk**	**High risk**
Control (*Y*=0)	*a*	*b*
Case (*Y*=1)	*c*	*d*

For *SNP*_*i*_ and *SNP*_*j*_ and each of 51 possible compositional epistasis models, the chi-square test statistic is calculated using Eq.(4). Those models with test statistics passing a given significance threshold will be considered as the possible interaction patterns of *SNP*_*i*_ and *SNP*_*j*_.

### Compositional epistasis detection in GWAS

In a typical GWAS, there are hundreds of billions of pairs of SNPs to be tested. It is computationally expensive to evaluate every possible compositional epistasis for all pairs of SNPs. However, it is widely believed that among the very large number of SNP pairs, only a small portion may be relevant with the disease trait. Therefore, it is a huge waste to test all SNP pairs to find significant compositional epistasis. If we can quickly compute the best fit of compositional epistasis model given the observed data for a SNP pair, we can first remove those pairs unlikely to be significant and then focus on evaluating all possible compositional epistasis model for the remaining SNP pairs. By doing so, the entire process will be substantially sped up. The approach in selecting the best splits for classification trees with categorical variables provides a solution to identify the compositional epistasis model best fitting the observe data.

In classification trees, leaves represent class labels, internal nodes represent features and branches represent conjunctions of features that induce class labels. In this work, class labels are phenotypes and features are genotypes. To construct a binary classification tree, a typical method iteratively searches all features for the best split. If the feature is categorical with *M* items, the number of all the possible splits is 2^*M*−1^. However, for a two-class classification problem, [14] proved the following theorem that reduces the search complexity into *O*(*M*).

**Theorem 1. ***Suppose there is a categorical variable X taking categorical values from* {1,2,⋯,*M*} *in two classes, class Y *= 0 and class *Y*= 1. *The categories are arranged in the ascending order of P*(*Y *= 1|*X *= i). *Then one of M − 1 splits, L *= {1,⋯,*m*} and *R *= {*m*+1,⋯,*M*} *where 1 ≤ m < M, minimize the misclassification rate.*

Theorem 1 only holds for the two-class problem. Some extensions to the multi-class problem have been proposed on the basis of Theorem 1 but they are only locally optimal.

In the test of compositional epistasis, we can re-arrange Table 3 into a 2×9 sorted ratio table (See Table 5). Then one of the 8 splits, *L*={1,⋯,*i*} and *R*={*i*+1,⋯,9} will lead to the minimum misclassification error. The intuition is straightforward. The best split should put all those categories leading to high probabilities of being in *Y*=0 into one side and the categories leading to high probabilities in *Y*=1 into another side. The connection between the misclassification rate of a split and the chi-square statistic of the corresponding 2×2 contingency tables is also simple. If the chi-square statistic of this table is small, then the risk factor, i.e. SNP, gives little information about the class because they are nearly independent. If the chi-square statistic is large, then the risk factor is very informative on class labels and certainly serves as a good predictor. In [15], it was shown that the split that leads to the minimum misclassification error also gives rise to the maximum of chi-square statistic of 2×2 contingency tables.

**Table 5:** The sorted ratio table for finding the maximum of chi-square statistics in the test of compositional epistasis

*Y*=0	*s*_1_	*s*_2_	*s*_3_	*s*_4_	*s*_5_	*s*_6_	*s*_7_	*s*_8_	*s*_9_
*Y*=1	*t*_1_	*t*_2_	*t*_3_	*t*_4_	*t*_5_	*t*_6_	*t*_7_	*t*_8_	*t*_9_
*Ratio*	*r*_1_	*r*_2_	*r*_3_	*r*_4_	*r*_5_	*r*_6_	*r*_7_	*r*_8_	*r*_9_

In this table, $r_{i}=\frac{s_{i}}{s_{i}+t_{i}}$ and *r*_1_≤⋯≤*r*_*i*_≤⋯≤*r*_9_.

Based on Theorem 1, we propose a two-stage (screening and testing) search method to find compositional epistasis in GWAS data. 

• In the screening stage, the method evaluates all SNP pairs by checking 8 splits to find an upper bound and remove pairs with the upper bound less than *τ*. The threshold *τ* corresponds to the significant threshold (with the Bonferroni correction) specified by users. Because the Bonferroni correction tends to be conservative, a smaller threshold can be used to put more SNP pairs into the testing stage. We set *τ*=20 in our method, which corresponds to the unadjusted *p*-value 7.744×10^−6^, which is a relatively liberal significance level for a genome-wide study.

• In the testing stage, the method checks each selected pair using all non-redundant compositional epistasis models. The *p* value for each model tested is adjusted by the Bonferroni correction, with the number of tests *L*(*L*−1)/2 where L is the total number of SNPs before screening.

### GPU implementation

To accelerate the analysis process in GWAS, the proposed method is implemented using the parallel computation of graphical processing units (GPUs) (http://docs.nvidia.com/cuda/). The development of GPUs enables modern display cards to have hundreds of core at a low price, which can be easily set up for the large-scale data analysis. To achieve a good speed-up, our GPU implementation maximizes the coalesced memory access and makes full use of the texture memory. The coalesced memory access groups 16 consecutive global memory transactions into a single memory transaction. It is the key technique to save memory access time in CUDA-enabled GPU. The texture memory is used for tasks with random memory access to improve the memory access speed. Our GPU implementation chooses the bit data structure and then fits the entire data into the GPU memory, which minimizes the overhead between the device and the host. The kernel program in our GPU implementation is designed with only a few registers being used and allows for a large number of concurrent threads. Without using GPU computing, our method needs around 120 hours to finish the genome-wide compositional epistasis analysis of a typical data set (with around 5,000 subjects and around 350,000 markers) on a single workstation. The GPU enabled implementation can finish the same analysis in two hours.

## Results

The compositional epistasis and statistical epistasis are two most commonly considered epistasis. In general, there are two types of statistical epistasis, named ‘Interaction’ and ‘Full Association’. In this section, we will evaluate these three types of epistasis using both simulated data and real data. To compare the statistical power among them, we have another issue of multiple test correction to consider. For each pair of SNPs, both the interaction test and the full association test compute one statistic and conduct the hypothesis test with the corresponding degrees of freedom. In the test of compositional epistasis, each SNP pair is associated with multiple epistatic patterns and thus with multiple statistics. In our comparison experiments, we choose the maximum one. Since we need to check 8 patterns to get the maximum statistic (see Theorem 1), we need to multiply the computed *P*-value with 8.

### Simulation 1: epistasis with main effects

#### Data generation

In this experiment, we select four epistasis models whose odds tables are given in Table 6. Please see [3] for the detailed description of these four models. For each model, we generate genotype data with the assumption that the SNPs satisfy Hardy Weinberg equilibrium in the general population with a given prevalence. We set the MAFs of disease associated SNPs as 0.1, 0.2, and 0.4. We generate the MAFs of un-associated SNPs uniformly from [0.05, 0.5]. The parameters of each model for each setting are calculated based on the pre-specified disease prevalence *p*(*D*) and the genetic heritability *h*^2^. The disease prevalence *p*(*D*) and genetic heritability *h*^2^ are computed as

(5) $$p(D)=\underset{i}{\sum }p(D|G_{i})p(G_{i}),$$

**Table 6:** The odds tables for four epistasis models

model 1	BB	Bb	bb	model 2	BB	Bb	bb
AA	*α*	*α*	*α*	AA	*α*	*α*(1+*θ*)	*α*(1+*θ*)
Aa	*α*	*α*(1+*θ*)	*α*(1+*θ*)^2^	Aa	*α*(1+*θ*)	*α*	*α*
aa	*α*	*α*(1+*θ*)^2^	*α*(1+*θ*)^4^	aa	*α*(1+*θ*)	*α*	*α*
model 3	BB	Bb	bb	model 4	BB	Bb	bb
AA	*α*	*α*	*α*(1+*θ*)	AA	*α*	*α*(1+*θ*)	*α*
Aa	*α*	*α*(1+*θ*)	*α*	Aa	*α*(1+*θ*)	*α*	*α*(1+*θ*)
aa	*α*(1+*θ*)	*α*	*α*	aa	*α*	*α*(1+*θ*)	*α*

The parameters *α* and *θ* control the prevalence *p*(*D*) (Eq.(5)) and the heritability *h*^2^ (Eq.(6)).

(6) $$h^{2}=\frac{\underset{i}{\sum }{\left(p(D|G_{i})-p(D)\right)}^{2}p(G_{i})}{p(D)(1-p(D))},$$

where *p*(*D*|*G*_*i*_) denote the probability of an individual being affected given its genotype combination *G*_*i*_ (i.e., the penetrance of *G*_*i*_). Let $p(\bar{D}|G_{i})$ denote the probability of an individual not being affected given its genotype *G*_*i*_. The odds of a disease for genotype *G*_*i*_ is defined as

(7) $$\text{OD}D_{G_{i}}=\frac{p(D|G_{i})}{p(\bar{D}|G_{i})}=\frac{p(D|G_{i})}{1-p(D|G_{i})}.$$

Then the penetrance *p*(*D*|*G*_*i*_) of the genotype *G*_*i*_ can be calculated using

(8) $$p(D|G_{i})=\frac{\text{OD}D_{G_{i}}}{1+\text{OD}D_{G_{i}}}.$$

**Figure 1:**
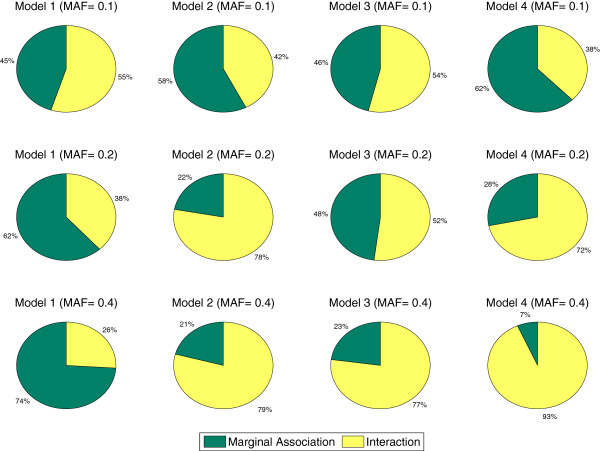
**Variance composition in the different epistasis models.** The total variance of disease traits is decomposed into two parts: the variance explained by marginal effects and the variance explained by interactions.

In our simulation, the prevalence *p*(*D*) and the heritability *h*^2^ are controlled by the parameters *α* and *θ* (see Table 6). We first specify the disease prevalence *p*(*D*), genetic heritability *h*^2^, and then numerically solve the parameters (*α* and *θ*) based on Eq.(5-8). For example, when *p*(*D*)=0.1 and *h*^2^=0.03 in model 1, we have *α*=0.1 and *θ*=3.45 for *MAF*=0.1. We simulate 100 data sets under each setting for each disease model. Each data set contains 1,000 SNPs. To take sample sizes into consideration, we generate 800 and 1,600 samples with the balanced design. Figure 1 provides the analysis of variance of the generated data. The total variance of disease traits is decomposed into two parts: the variance explained by individual main effects and the variance explained by interactions, i.e. epistasis.

#### Performance comparison

**Figure 2:**
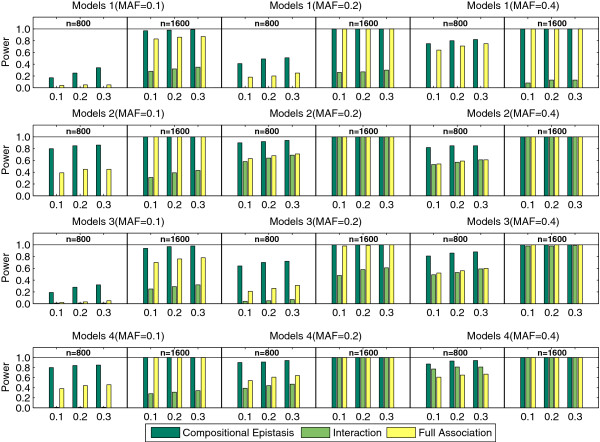
**The performance comparison of three epistasis tests.** The significance thresholds are selected as 0.1, 0.2 and 0.3 after the Bonferroni correction.

The performance comparison of three tests is provided in Figure 2 with the significance thresholds selected as 0.1, 0.2 and 0.3 after the Bonferroni correction. It exactly matches the analysis of variance (ANOVA) of the four disease models. It is not surprising to see that the test of compositional epistasis and the test of full association outperform the test of interaction because most models display noticeable main effects. Specifically, when the MAF is high, which indicates that all the *p*_*ij*_ in Table 1 are non-trivial, the test of compositional epistasis and the test of full association perform equally well. However, when the MAF is low, the test of interaction and the test of full association perform poorly while the test of compositional epistasis performs reasonably well. For all the models, the test of compositional epistasis has a higher power than the other two tests. We can also see that the sample size plays an important role for all methods. The power increases significantly when the sample size increases from *n*=800 to 1600. In general, the test of interaction’ has the good performance in epistasis models in which marginal effects of SNPs are trivial. The test of full association has its advantage in epistasis models that own both marginal effects and interaction effect. The test of compositional epistasis has high power in the situation that a sparse contingency table is involved in the epistasis test due to the low MAF of loci.

### Simulation 2: epistasis without main effects

**Figure 3:**
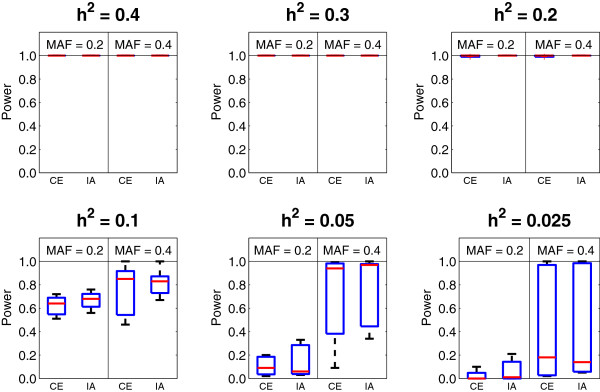
The power comparison between the compositional epistasis (CE) and the interaction (IA) in models without main effects.

This type of epistasis demonstrates weak main effects, but strong interaction effect. Finding such type of epistasis is a challenging task. It is the advantage of the interaction test to detect such type of epistasis. We use the commonly used data sets from the Dartmouth Medical School in this experiment. The web-site, http://discovery.dartmouth.edu/epistatic_data, provides 70 models, composed of combinations of the following parameter values: (1) two MAF settings of 0.2 and 0.4; (2) six heritability settings of 0.4, 0.3, 0.2, 0.1, 0.05 and 0.025. For each model, the statistical power is evaluated under different sample sizes, including 400, 800 and 1600, where there are equal numbers of cases and controls. For each setting, 100 data sets are generated. Each data set contains 1000 SNPs. Figure 3 summarizes the comparison results for the 70 models categorized with the heritability. It can be observed that for epistasis without main effects, the test of compositional epistasis and the test of interaction perform equally well.

### Simulation 3: type-1 error rate

**Figure 4:**
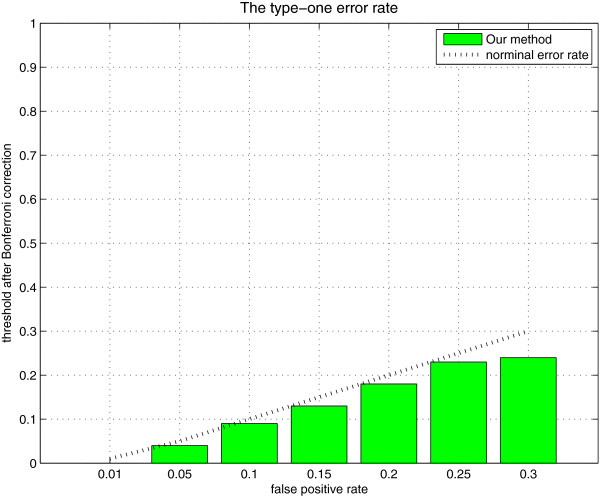
The type-I error rates in null simulation.

To show the type I errors of our method, we conduct the following null simulation. We generate 100 null data sets. Each data set contains 2,000 SNPs and 2,000 samples. All SNPs are generated independently with MAFs uniformly distributed in [0.05,0.5]. The result is shown in Figure 4. It can be seen that the type I errors of our method is close to the nominal level.

### Experiments on seven data sets from WTCCC

The Wellcome Trust Case Control Consortium (WTCCC) is a collaboration of many British research groups. In the first phase, the WTCCC has examined the genetic signals (500K SNPs) of seven common human diseases: bipolar disorder (BD), coronary artery disease (CAD), Crohn’s disease (CD), hypertension (HT), rheumatoid arthritis (RA), type 1 diabetes (T1D), and type 2 diabetes (T2D) (14,000 cases in total and 3,000 shared controls). Before we analyze these data sets, we first apply a similar quality control procedure as suggested in (WTCCC, 2007) to pre-process the data. Next we filter out those SNPs with significant individual effects. The threshold is chosen as *p*=3.059×10^−7^, which is equivalent with *p*_*c*_=0.10 after the Bonferroni correction. The number of remaining SNPs is roughly 350,000 for each disease. The results from the three epistasis tests are reported in Table 7.

**Table 7:** The number of SNP pairs identified from the WTCCC data sets of seven diseases under different tests

	**BD**	**CAD**	**CD**	**HT**	**RA**	**T1D**	**T2D**
Compositional Epistasis	0	0	17	0	47	234	3
Interaction	0	0	1	0	0	317	0
Full Association	0	0	0	0	10	346	0

#### T1D

**Figure 5:**
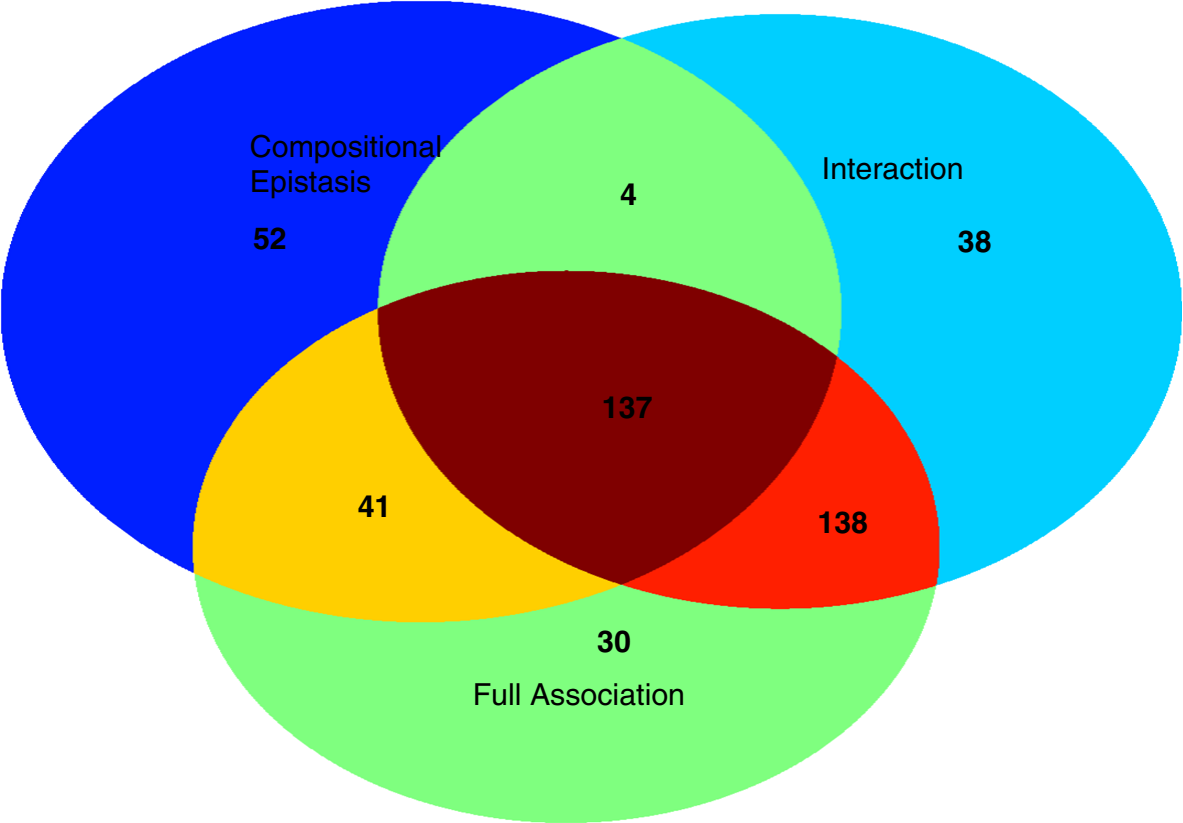
The distributions of SNP pairs among three epistasis tests in T1D.

**Figure 6:**
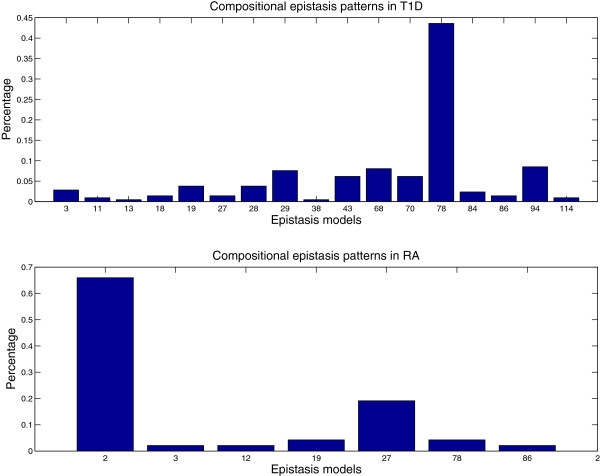
Compositional epistasis patterns in T1D and RA.

For T1D, all identified SNP pairs by three epistasis tests are located in the major histocompatibility complex (MHC) regions. The MHC region in chromosome 6 has long been comprehensively studied for many decades because its high diversity and significance in infection, inflammation, autoimmunity, and transplant medicine [16]. The recent study conducted by the WTCCC [17] has shown that T1D are strongly associated with the MHC region via single-locus association mapping. The epistasis analysis provides extra evidence for the association study. Please note that the SNPs involved in the identified SNP pairs do not display significant individual effects and thus can not be reported by the single-locus test. The distributions of SNP pairs among three epistasis tests in T1D are visualized in Figure 5. A further analysis reveals that 44 percentage of the identified SNPs pairs possess an XOR pattern (M78). The top panel of Figure 6 provides all identified compositional epistasis patterns in T1D. This is a new finding and may provide some new insights in studying the causes of T1D.

#### RA

For RA, the test of compositional epistasis reports 47 pairs, which includes the 10 pairs reported by the test of full association. The test of interaction does not report any significant pairs. A careful inspection of these pairs reveals that the epistatic effect of these pairs consists of partial individual effects and partial interaction effects. Among 47 reported pairs, 43 pairs involve SNP rs2107191 and the paired SNPs are all located in a very gene-rich region (the genome location is from 29,778,109 to 30,363,351). There are about 31 pairs involving SNP rs2107191 displaying a recessive-interference pattern (M2) [13]. The SNP rs2107191 is located very closely with gene OR2H1, which has been reported as a susceptibility locus for RA [18]. The bottom panel of Figure 6 provides all identified compositional epistasis patterns in RA. It can be observed that T1D and RA have different epistasis patterns. A further investigation on these patterns may reveal a new direction on the study of the etiology of RA and T1D.

## Discussions

In this work, we have focused on the genome-wide case-control studies; i.e., the disease phenotype can be represented as a binary variable. In its current testing, the compositional epistasis can not be easily extended to consider continuous phenotypes. Moreover, the current work only detect two-way compositional epistasis. However, we note that there is no widely accepted definition of high-order compositional epistasis. These issues are worth pursuing in the future.

## Conclusions

Studying the epistasis between two loci is a natural step following traditional and well-established single locus analysis. In this paper, we have proposed a computationally efficient and statistically sound method to test compositional epistasis in GWAS data. The method is applicable to case-control studies and consists of a two-step (screening and testing) process. In the screening stage, only a limited number of epistatic patterns are evaluated for each pair of SNPs and those passing a specified threshold are selected to be more thoroughly studied in the testing stage, where all epistatic patterns for each selected pair are evaluated. The method is implemented using the parallel computational capability of commercially available GPUs to greatly reduce the computation time involved. We have successfully applied our method to analyze seven data sets from the WTCCC. Our experimental results demonstrate that the complete compositional epistasis detection is computationally feasible in GWAS.

## Competing interests

The authors declare that they have no competing interests.

## Authors’ contributions

XW and CY designed the models and simulation studies. QY and WY initialized the study and proposed the modeling framework. YH and WY directed the evaluation of methodologies. All authors contributed to the writing of the manuscript. All authors read and approved the final manuscript.
